# Laboratory Diagnosis of Cutaneous and Visceral Leishmaniasis: Current and Future Methods

**DOI:** 10.3390/microorganisms8111632

**Published:** 2020-10-22

**Authors:** Juliana Quero Reimão, Elizabeth Magiolo Coser, Monica Ran Lee, Adriano Cappellazzo Coelho

**Affiliations:** 1 Departamento de Morfologia e Patologia Básica, Faculdade de Medicina de Jundiaí, Jundiaí 13202-550, Brazil; juliana_reimao@yahoo.com.br (J.Q.R.); m0nicalee@hotmail.com (M.R.L.); 2 Departamento de Biologia Animal, Instituto de Biologia, Universidade Estadual de Campinas (UNICAMP), Campinas 13083-862, Brazil; elizabethmcoser@hotmail.com

**Keywords:** leishmaniasis, cutaneous leishmaniasis, visceral leishmaniasis, *Leishmania*, diagnosis

## Abstract

Leishmaniasis is a neglected tropical disease with two main clinical forms: cutaneous and visceral leishmaniasis. Diagnosis of leishmaniasis is still a challenge, concerning the detection and correct identification of the species of the parasite, mainly in endemic areas where the absence of appropriate resources is still a problem. Most accessible methods for diagnosis, particularly in these areas, do not include the identification of each one of more than 20 species responsible for the disease. Here, we summarize the main methods used for the detection and identification of leishmaniasis that can be performed by demonstration of the parasite in biological samples from the patient through microscopic examination, by in vitro culture or animal inoculation; by molecular methods through the detection of parasite DNA; or by immunological methods through the detection of parasite antigens that may be present in urine or through the detection of specific antibodies against the parasite. Potential new methods that can be applied for laboratory diagnosis of leishmaniasis are also discussed.

## 1. Introduction

Leishmaniasis is a parasitic disease caused by the parasite protozoan of the genus *Leishmania* that are transmitted to humans by the bite of a female sand fly vector. The parasite alternates between flagellated promastigotes in the insect vector and as intracellular amastigotes in the mammalian host. At least 20 species of parasite are responsible for visceral and cutaneous leishmaniasis in humans with clinical features of each form of the disease depending on the species of *Leishmania* and the immune response of the host [1,2]. This neglected tropical disease is found in all continents, except Oceania, including areas in Northeastern Africa, Southern Europe, Asia, and Latin America and affects almost one hundred countries [3]. The number of cases reported globally ranges from 0.2 to 0.4 million cases for visceral leishmaniasis (VL) and 0.7 to 1.2 million cases of cutaneous leishmaniasis (CL) per year, with an estimate of at least 20,000 deaths per year [3]. Only six countries suffer more than 90% of VL cases worldwide: India, Bangladesh, Sudan, South Sudan, Ethiopia, and Brazil, while CL is reported in a higher number of countries and almost 75% of cases occur in ten countries: Afghanistan, Algeria, Colombia, Brazil, Iran, Syria, Ethiopia, Sudan, Costa Rica, and Peru [3]. For CL, an increase in the incidence in the Middle East and Americas has been reported over recent years, particularly due to conflict and environmental factors [4].

Visceral leishmaniasis is the systemic form of the disease that affects mainly the liver, spleen, and bone marrow and may be lethal if not treated. It is caused by *Leishmania* (*Leishmania*) *donovani* in Northeastern Africa and Southeastern Asia and *L*. (*L*.) *infantum* in Southern Europe, Northeastern Africa, and Central and South America [2,5]. This clinical form of the disease has a zoonotic transmission in the case of *L*. (*L*.) *infantum* (from animal to vector to human) and an anthroponotic transmission (from human to vector to human) that is caused by *L*. (*L*.) *donovani*, although animal reservoirs, including dogs, have already been reported as hosts for this species [2,5,6]. Post-kala-azar dermal leishmaniasis (PKDL) is a complication of VL generally observed in post-treatment patients and immunosuppressed individuals infected by *L*. (*L*.) *donovani* and it is characterized by macular, papular, or nodular lesions on the skin [7]. Around 5–10% of patients with VL develop this dermal manifestation that is mainly associated with incomplete treatment [8]. Most of the cases occur in Southeast Asia (India, Nepal, and Bangladesh) and East Africa (mainly Sudan) [7]. In addition to this, patients infected with VL may not develop clinical symptoms and may remain infected at a sub-clinical level, increasing the challenge of a correct diagnosis and appropriate treatment and consequently restricting the control of the disease in endemic areas [9].

The localized CL is characterized by a papule that develops at the site of the bite of the sand fly that enlarges to a nodule and then ulcerates [2]. In 10% of cases, CL may progress in more severe manifestations: mucocutaneous leishmaniasis (ML), diffuse CL (DCL) and disseminated CL [2]. These different clinical manifestations of CL are caused by zoonotic species of *Leishmania*, with the exception of *L*. (*L*.) *tropica* that is also considered an anthroponotic species [10]. Mucocutaneous leishmaniasis is characterized by disfiguring and destructive lesions of the oronasopharyngeal mucosa, due to a strong immunopathological response and is caused by *L*. (*Viannia*) *brazilensis* and also *L*. (*V*.) *guyanesis* [2]; while DCL is caused by *L*. (*L*.) *amazonensis*, *L*. (*L*.) *mexicana* and *L*. (*L*.) *aethiopica* and is characterized by nodules spread across the body of the patient that do not ulcerate, in an immune status of absence of cellular response [2,11]. Finally, in the disseminated CL, the patient presents several pleomorphic lesions, with acneiform and papular aspect, distributed in noncontiguous areas of the body. Cases of disseminated CL have been correlated to *L*. (*V*.) *braziliensis* [12,13]. Moreover, similarly to VL, CL patients may not develop clinical symptoms either and the disease at a sub-clinical level may restrict the control of disease [9].

*Leishmania*/HIV co-infection cases have been reported for VL, PKDL, and CL patients, with an increasing number of cases worldwide due to the spread of the AIDS pandemic from urban to rural regions, being considered one of great challenges for leishmaniasis control [2,5,14,15,16,17,18]. In Europe, cases of VL/HIV co-infection have been noted in intravenous drug users through the sharing of needles [15]. Atypical clinical cases are recurrent in VL and CL patients co-infected with HIV, and more severe manifestations have also been reported [16,18]. In CL/HIV co-infected patients from Brazil, an extensive variety of lesions and ulcerations have been described [18], while in VL/HIV patients, parasites have been detected in organs that are not usually found in non-immunosuppressed patients, such as, for example, the gastrointestinal mucosa and respiratory tract [2,19]. HIV-infected patients have an increased risk of being infected by the parasite, and in the case of co-infection, the treatment may be compromised due to the role of CD4^+^ T cells in the clearance of intracellular amastigotes [20].

There is a limited number of drugs available for chemotherapy of leishmaniasis. Pentavalent antimonials, amphotericin B and pentamidine have been used against the parasite for several decades, while miltefosine and paromomycin have been approved for treatment more recently [21]. In general, the use of these drugs does not take into consideration the diversity of clinical forms of the disease, in part because the diagnosis methods for species discrimination are still limited in areas where more than one species is endemic.

The numerous clinical manifestations of the disease mean the diagnosis of current and previous cases of infection is still a challenge for the correct identification of the etiological agent of the disease. Lesions in CL, for example, may vary greatly in relation to the size, clinical appearance and period of evolution and spontaneous cure. Clinical findings in patients with CL due to *L*. (*V*.) *braziliensis* or *L*. (*V*.) *guyanensis* have already been associated with a particular aspect of skin lesions related to number, size, location and pattern of lymphatic involvement [22]. Patients infected by *L*. (*V*.) *guyanensis* had smaller and more numerous lesions, located in most of the cases above the waist, while in infections due to *L*. (*V*.) *braziliensis*, patients had fewer lesions that were located mainly on the lower limbs [22]. In addition, differential diagnosis is essential because other diseases may have clinical aspects similar to CL (e.g., leprosy, keloid, skin cancers, tuberculosis, cutaneous mycoses) and VL (e.g., Chagas disease, hepatosplenic schistosomiasis, amoebic liver abscess, disseminated histoplasmosis, mononucleosis, hepatitis, tuberculosis, malaria, sickle cell anemia, lymphoma, chronic myeloid leukemia, systemic lupus erythematosus, and liver cirrhosis, among others) [5,9,12]. In regions where the disease is endemic, the diagnostic tests available may be limited and depend more on the resources and infrastructure available than the accuracy of the employed method.

**Figure 1:**
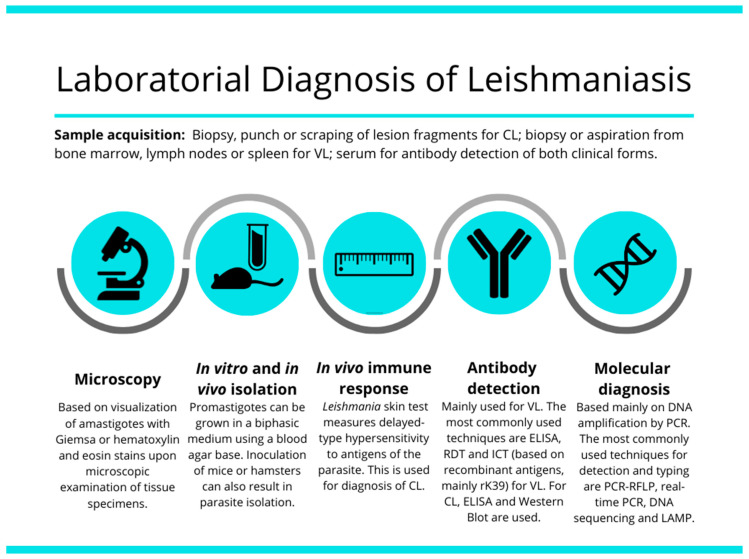
Illustrative image showing the most used tools and strategies for VL and CL laboratorial diagnosis.

In this review, we describe the main methods available for detection and identification of CL and VL, and their current limitations and challenges. Current diagnostic methods include the parasitological, immunological and molecular methods. The broad spectrum of clinical manifestations in both main forms of disease makes differential diagnosis an essential goal to the current identification of the etiological agent of this spectrum of diseases. Challenge for the current laboratory methods of diagnosis are the detection of the etiological agent and the correct identification of the species responsible for the clinical form of the disease. Light-microscopic examination of tissue smears or sections (histopathology), in vitro culture, serologic testing, and molecular tests based on DNA detection and amplification are the most used options for leishmaniasis diagnosis [23] (Figure 1), as discussed below.

## 2. Parasitological Methods for Diagnosis of Leishmaniasis

The parasitological methods are broadly practiced in leishmaniasis diagnosis, based on visualizing *Leishmania* amastigote forms in infected tissues [24]. While these techniques are highly specific for diagnosing leishmaniasis, they are insufficiently sensitive [25]. Moreover, these methods do not allow for discrimination between the different species of *Leishmania*. In CL, the sample is acquired through biopsy, punch, or scraping from mucosa or skin lesions. In skin lesions, the use of scrapings and cytology brushes is recommended instead of the more invasive biopsy [25]. The obtained sample stained with Giemsa proceeds to a histopathological exam, where amastigotes are identified through microscopical analysis. Moreover, the press-imprint-smear method is recommended since it is a quick, low cost, and relatively sensitive method. For the imprint, the biopsy sample is put on a glass slide, and another glass slide is used to cover the tissue fragment in the form of a sandwich. On a firm surface, the tissue fragment between both glass slides is squeezed. Pressure is applied to the middle of the slides causing the juice and tissue cells to spread out across both slides’ surfaces that are in contact with the sample. The smears are then air dried, fixed in methanol, stained with Giemsa, and examined microscopically using a 100× oil immersion lens [26].

The parasitological exam is the current gold standard for diagnosis of VL, in which the visualization of the amastigote form of the parasite in the biopsied material is the confirmatory diagnosis [27]. The biopsy should preferably be obtained from the bone marrow, since the procedure is more secure, or from the lymph nodes or spleen. Spleen biopsy must be performed in a hospital environment and under surgical conditions, as the spleen aspiration can be complicated by life-threatening hemorrhaging (0.1%) amongst individuals associated with severe thrombocytopenia [28]. Sensitivity will vary according to the biopsied tissue, with spleen aspiration biopsy having the highest sensitivity, from 93% to 99% [27], followed by bone marrow aspiration, liver biopsy and lymph node aspiration. Furthermore, the obtained material can also be purposed for smears and examination after Giemsa staining.

### 2.1. Microscopic Examination

The microscopic exam is the most widely available test for the diagnosis of leishmaniasis, but it does not allow the identification of *Leishmania* species [23]. For CL, the direct examination of stained material removed from the lesion (obtained by biopsy, punch, scraping, smear or imprinting) using light microscopy, has a 50–70% sensitivity for *Leishmania* species from Africa, Asia and Europe, and 15–30% for species from the Americas (Table 1) [16,25,26,29,30]. For samples from CL patients, a combination of different methods is recommended, given the limited sensitivity of these methods, particularly for samples of ML that have a low parasite load. Combination with PCR, for example, may increase sensitivity to more than 80% [31,32]. For VL, aspirates from the lymph nodes, bone marrow, or spleen are the main sources of samples for microscopic examination with relative variations in sensitivity; the aspirate of spleen being the sample with the highest sensitivity for microscopic analysis [5,33,34]. In the human host, only the amastigotes stage is seen through microscopic examination of tissue specimens, which can be visualized with both Giemsa and hematoxylin and eosin stains [23]. *Leishmania* amastigotes are intracellular round or oval bodies, about 2–4 μm in diameter, with characteristic nuclei and kinetoplasts [35].

### 2.2. In Vitro Cultivation of Leishmania

An alternative to increasing sensitivity through direct examination is to use part of the biopsied material for inoculation in a culture medium, although this is rarely used in routine clinical practice (Table 1) [19]. Isolation followed by in vitro cultivation is useful for confirmation of *Leishmania* infection and for species identification by molecular methods (see below).

For isolation purposes, the culture medium should mimic the conditions of the vector insect to prompt viable amastigotes to differentiate promastigotes and reproduce [36]. Promastigotes can be grown in some media already established for cultivation of parasites at temperatures below 28 °C (generally between 22 °C and 26 °C) [37]. The main media used for the cultivation of promastigotes are the biphasic medium, Nicolle’s modification of Novy and MacNeal’s medium (NNN), and Schneider’s Drosophila medium supplemented with fetal bovine serum [37]. In these media, promastigotes are noted after a period of 7–21 and 2–7 days, after inoculation of amastigotes into NNN and Schneider’s medium, respectively. Sensitivity of in vitro cultivation may also be dependent on the species of parasite. In skin aspirates, the sensitivity from samples of patients infected by *L*. (*V*.) *braziliensis* and *L*. (*V*.) *guyanensis* was of 47% and 91.2%, respectively [22]. In vitro parasite growth in a culture medium takes several days and can eventually be contaminated with bacteria or fungi. Additionally, the results may vary depending on the parasite burden in the biopsy sample, the expertise of the laboratory technician and the culture media used [12,37].

More recently, a microculture technique has been developed as an alternative for parasite cultivation. This technique uses the scraping of a cutaneous lesion for cultivation in a capillary tube in sterile conditions [38]. This method is cost-effective and more sensitive than the standard cultivation, does not require the use of a needle and syringe, and can also be used for isolating parasites for drug susceptibility assays and typing. In the microculture technique, promastigotes can be noted after 2–3 days and sensitivity and specificity were 94% and 100%, respectively [38].

### 2.3. Inoculation of Leishmania in Experimental Animals

This method consists of the inoculation of parasites obtained from biological samples from patients into the footpad, nose, or tail base of mice (for CL), or intravenous or intraperitoneal inoculation in mice or golden hamsters (for VL) (Table 1). Interestingly the development of the disease was faster in hamsters infected with *L*. (*V*.) *guyanensis* than in those infected with *L*. (*V*.) *braziliensis*, indicating that this diagnostic approach may be different depending on the species of parasite [22]. Inoculation in animals is a time-consuming procedure and require trained professionals to perform the procedure [19,35]. Inoculation of parasites in animals is restricted solely to research centers that contain animal facility to house stock, breed and perform experimental infection. This method is not considered a first diagnostic procedure, as several weeks are required for confirmation of the presence in the infected animals, but is an important complementary tool, especially in dubious cases [19].

### 2.4. Xenodiagnosis

Xenodiagnosis is a method that uses the insect vector of a pathogen as a culture medium for the detection of infection in a mammalian host/patient [40]. In Chagas disease, xenodiagnosis is a classical method that documents the presence of *Trypanosoma cruzi* by exposing possibly infected blood of the patient to nymphs of the triatome bugs and then examining the vector for the presence of the parasite it may have ingested [99]. This method can be performed in two distinct ways: direct or indirect xenodiagnosis. In direct xenodiagnosis, an uninfected hematophagous insect feeds on a suspected individual while in the indirect method, the insect feeds on heparinized blood through a feeder membrane (e.g., chicken skin) [40]. The latter method avoids the risks of hypersensitivity to insect bites and transmission of other infectious agents. Xenodiagnosis was adapted for experimental leishmaniasis using mice infected with *L*. (*L*.) *donovani* as a model [100] and was recently applied for diagnosis on VL and PKDL patients, by the direct and indirect procedures that were infectious to the sand fly *Phlebotomus argentipes* (Table 1) [39]. Direct xenodiagnosis was significantly more sensitive than the indirect xenodiagnosis and parameters associated with positive xenodiagnosis were nodular lesions, parasite burden and positive microscopy skin [39]. It would be interesting to investigate the potential for detection of this method in infected, cured, and asymptomatic patients, particularly those with CL.

As other parasitological methods, this method does not discriminate *Leishmania* species and is time-consuming (requiring 2–5 days to get final results). Additionally, it requires a well-established sand fly rearing laboratory and a trained entomologist for dissection of the sand flies for microscopic examination of *Leishmania* infection [40,101].

## 3. Molecular Methods for Diagnosis of Leishmaniasis

Several molecular methods have already been evaluated for diagnosis of leishmaniasis. PCR-based assays are the basis for *Leishmania* detection and typing (Table 1). They have been considered the main method for molecular diagnosis of leishmaniasis. PCR-based assays are highly recommended for species typing, particularly for species responsible for CL that may be caused by different species of the parasite. In Brazil, for example, at least seven species may cause CL. The advantage of using PCR-based methods are, in general, their feasibility, safety, and reliability for application in a routine laboratory [102]. As an alternative for species typing, there is multilocus enzyme electrophoresis (MLEE) based on the profile of proteins of a specific species (see below). Recently, mass spectrometry was also proposed as an alternative for species identification of *Leishmania* [47].

### 3.1. Multilocus Enzyme Electrophoresis (MLEE)

This method is completely dependent on isolation of the parasite followed by its cultivation and is based on the profile of a set of proteins in a pH-dependent gel electrophoresis. The classification of each species is determined by the combination of these proteins (described as zymodeme) (Table 1) [41]. MLEE is considered to be the gold standard for *Leishmania* typing by the World Health Organization (WHO) and it is maybe the only method available that is able to discriminate almost all the species responsible for human leishmaniasis [52]. MLEE also requires a high level of technical expertise of the professionals and is extremely time-consuming. Based on the reference strains of the parasite, the MLEE method covers variations in the genus *Leishmania*, but its use is limited to a restricted number of laboratories worldwide.

### 3.2. Monoclonal Antibodies

The use of monoclonal antibodies was also established for identification of *Leishmania* species, and similarly to MLEE, requires isolation followed by cultivation of parasites as promastigotes. The most extensive study, for species discrimination, reported the generation of monoclonal antibodies against almost all species endemic in Americas of both subgenera (Table 1) [42]. Monoclonal antibodies specific to species endemic in Europe, Asia, and Africa were also generated and are available for *L*. (*L*.) *major*, *L*. (*L*.) *donovani*, and *L*. (*L*.) *tropica* [43,44,45]. This method is rarely applied in the clinical context, since the procedure is time-consuming, and requires highly trained professionals and standardization. Moreover, its use is restricted to parasites obtained from clinical samples.

### 3.3. Mass Spectrometry (MS)

Matrix-assisted laser desorption ionization time-of-flight (MALDI-TOF) MS has already been proposed as an alternative for identification of prokaryotic and eukaryotic unicellular organisms [103,104]. MALDI-TOF MS was also implemented for species identification of the genus *Leishmania* (Table 1) [46,47,48]. This approach was applied using cultivated *Leishmania* promastigotes from a 56-reference database, that was then used for typing a panel of 69 isolates obtained from patients, where 66 were correctly identified [46]. This approach was later improved through the construction of a reference mass-spectral library for *Leishmania* identification using 33 species of 10 complexes that were then tested with an independent panel of 268 samples from different sources [47]. Among isolates, only one was misidentified at the complex level (typed as *L*. (*V*.) *guyanensis* instead of *L*. (*V*.) *braziliensis*), and the other 30 misidentifications were limited to the following complexes: *L*. (*V*.) *braziliensis* (*L*. (*V*.) *braziliensis*/*L*. (*V*.) *peruviana*), *L*. (*V*.) *guyanensis* (*L*. (*V*.) *guyanensis*/*L*. (*V*.) *shawi*/*L*. (*V*.) *panamensis*), and *L*. (*L*.) *donovani* (*L*. (*L*.) *donovani*/*L*. (*L*.) *infantum*/*L*. (*L*.) *archibaldi*) [47]. This method proved to be a potential tool for species typing, and was faster compared to other molecular methods (if considered that the parasite was already isolated). Unfortunately, there is the need of a fully-equipped laboratory containing costly equipment and professionals trained in the performance of MALDI-TOF MS analyses. Finally, similar to MLEE and the use of monoclonal antibodies for typing, MALDI-TOF MS also requires isolation and cultivation of the parasite for species identification.

### 3.4. PCR-Based Methods

Several PCR-based methods have a level of sensitivity sufficient for the detection and typing of *Leishmania* species. The main advantage of PCR-based approaches is that they do not require parasite cultivation and may be directly applied to clinical samples. PCR-based approaches are based on coding or non-coding regions of the nuclear and mitochondrial genomes of the parasite. The PCR product consists of a specific amplification of the target DNA evaluated on a conventional agarose gel followed by downstream analysis, such as through the use of restriction endonucleases, hybridization, or DNA sequencing [52,105], or by detection and analysis of fluorescent signals during amplification in a real-time PCR apparatus (Table 1). The signal is generated mainly by the use of intercalating fluorescent dyes (e.g., SYBR Green) or fluorescent probes (e.g., TaqMan^®^) [106,107] (see below). Beyond detection and typing of the parasites, a quantitative PCR may also be useful for monitoring clinical cure and follow-up of patients [108].

Alternatively, a specific PCR product targeting single or multiple species is also available, and, in general, they are able to identify subgenera and/or a group of species [52]. More recently, loop-mediated isothermal amplification (LAMP) has also been validated as an alternative method for diagnosis (Table 1) [66,109]. Finally, for all these methods, standardization and optimization are required for use in hospitals and reference centers. To achieve reliable results, a standardized genomic DNA extraction protocol, internal controls for PCR reaction and genomic DNA of reference strains as control are all highly recommended. Evaluation by an external quality control program is also recommended [16,110].

#### 3.4.1. PCR-RFLP (Restriction Fragment Length Polymorphism)

This approach is based on the pattern of DNA fragments after digestion with one or some restriction enzymes and then evaluated in gel electrophoresis. The technique is relatively simple and can be performed in a laboratory with a PCR machine. The main targets used for this method are rDNA locus (ITS1 and ITS2), the heat shock protein 70 (hsp70) gene, miniexon for nuclear DNA, and minicircles for kinetoplast DNA (kDNA) (Table 1) [9,52].

The rDNA *locus* in *Leishmania* has around 10–20 repeated copies in tandem in a single chromosome of the parasite [111]. Each unit of repetition of around 12.5 kb contains the ribosomal genes and spacers. Using rDNA as a target, ITS1 and 5.8S were applied for evaluation of PCR-RFLP using the *Hae*III restriction enzyme for species discrimination. This method was evaluated in clinical samples and was useful for discrimination of the subgenus *Leishmania* [112]. Although divergent for discrimination of species of the subgenus *Viannia* by DNA sequencing, this PCR-RFLP protocol is unable to discriminate these species. This is possible when using ITS1, 5.8S, and ITS2, a region termed as IRT (intergenic region typing), for species discrimination of this subgenus, although several restriction enzymes need to be used [113]. Finally, due to the high level of conservation, 18S (or SSU) is not useful for species discrimination [114].

The *hsp70* gene has been extensively studied and validated as a potential target for species discrimination by the PCR-RFLP approach. This gene of 5–10 copies per haploid genome was initially evaluated as a potential marker for discrimination of species of the subgenus *Viannia* [115]. Further studies used other restriction enzymes and amplified fragments targeting this gene that were also described as useful for species discrimination [116,117,118]. On the other hand, this target should be used with care, particularly for some species of subgenus *Viannia* that may have the same profile of restriction with the *Hae*III enzyme (*L*. (*V*.) *guyanensis* and *L*. (*V*.) *lindenbergi*), whilst different strains and isolates of the same species may have different profiles with the same restriction enzyme, leading to misidentification [119]. Alternatively, *Leishmania* typing using the *hsp70* gene may be solved by DNA sequencing [119] or HRM [57] (see below).

The miniexon gene is a multicopy gene distributed in tandem in *Leishmania* (100–200 copies), making this *locus* a potential target for parasite detection directly in clinical specimens [120]. Both exon and intron sequences are conserved and only the intergenic region of miniexon genes may be an alternative for discrimination of some species of *Leishmania* by PCR amplification [121,122]. The employment of a RFLP approach to this target increases the number of species that can be identified [120,123]. This method was not evaluated in *L*. (*V*.) *lainsoni* and *L*. (*V*.) *lindenbergi* and cannot discriminate *L*. (*V*.) *guyanensis*/*L*. (*V*.) *panamensis* and *L*. (*V*.) *braziliensis*/*L*. (*V*.) *peruviana* [120,123]. Later, it was demonstrated that sequencing of the amplified fragment is more informative than a restriction profile that needs up to five restriction enzymes for species discrimination [124].

Coding genes of antigens, mainly the metalloprotease glycoprotein 63 (GP63) and cysteine proteinase B (CPB), were also validated for *Leishmania* typing [52]. Both are multicopy genes present in tandem in the *Leishmania* genome, offering sensitivity for application in clinical samples without the need for isolation [52]. Although validated in the main endemic species responsible for the clinical forms worldwide, there is still no study using these targets as a universal protocol to describe *Leishmania* species by PCR-RFLP [125,126]. Interestingly, a combination of multiple targets (*gp63*, *hsp70*, *cpb*, and *h2b* (histone H2B)) for typing using a PCR-RFLP analysis proved to be a robust method for the typing of strains and isolates of *Leishmania* endemic in the Americas [127].

Mitochondrial DNA of *Leishmania* consists of approximately 10,000 copies of minicircles of 0.5–2 kb and around 20–40 copies of maxicircles of approximately 20–40 kb, termed as kinetoplast DNA (kDNA) [128]. These two classes of molecules contain genes that encode mitochondrial genes (in maxicircles) and small RNAs, termed guide RNAs (in minicircles). The elevated number of copies of both classes of these circular DNAs allows their use as target for detection of the parasite directly from clinical sample. Polymorphisms in size of minicircles were investigated for identification and discrimination of *Leishmania* species. Although some differences occur in the size of the variable region of minicircles in *Leishmania* species, some species have a similar size, restricting their use for species discrimination of both subgenera [129,130]. Moreover, these studies were performed essentially in reference strains and variability in minicircles may be found in clinical isolates of the same species, hindering its use for species discrimination [52,54].

#### 3.4.2. DNA Sequencing

This is based on technology described by Sanger et al., [131] that uses the incorporation of chain-terminating dideoxynucleotides for the determination of the nucleotide sequence of a determined fragment of DNA, being highly useful for identification of *Leishmania* species. In addition, DNA sequencing may be useful for phylogenetic studies [9]. By means of this method, typing of *Leishmania* species may be performed by analysis of single nucleotide polymorphisms (SNPs) or by comparison of the sample’s sequence with reference sequences of *Leishmania* spp. in a dendrogram [52]. DNA sequencing of PCR-amplified products has been applied to several targets of the genome of the parasite for species identification. It has been described as useful for species discrimination using several nuclear and kDNA targets. The main targets used for DNA sequencing are rDNA (18S, ITS1 and ITS2), *hsp70*, miniexon, 7SL RNA, genes encoding metabolism enzymes for nuclear DNA and cytochrome B and minicircles for kDNA (Table 1) [9,52].

In the rDNA *locus*, the main regions useful for detection and typing species of parasite are 18S, ITS1 and ITS2. In the case of 18S, the limitation is its high level of conservation, preventing its capacity for species discrimination; although it may be useful for discrimination of genus *Leishmania* from other trypanosomatids and human genomic DNA [114,132]. On the other hand, ITS regions are highly variable among species of both subgenera, allowing the correct typing of *Leishmania* species by DNA sequencing [133,134].

The *hsp70* gene sequence has also been used for species discrimination, whilst it can also be used for studies involving taxonomy and phylogeny [52,134,135]. SNPs in the coding sequence of this gene are enough for discrimination of *Leishmania* species of both subgenera, including species less prevalent in endemic areas of South America, such as *L*. (*V*.) *naiffi* and *L*. (*V*.) *lindenbergi* [119,134,135,136].

The miniexon gene is conserved and is not useful for species discrimination by DNA sequencing [137]. On the other hand, the non-transcribed spacer is highly variable in sequence and size, allowing discrimination of the *Leishmania* subgenus and most species of the subgenus *Viannia* (*L*. (*V*.) *lainsoni* and *L*. (*V*) *lindenbergi* were not evaluated) [134]. Another alternative is the gene encoding 7SL RNA that is present in several copies in the genome of the parasite. It has a divergent domain making it a potential target for detection and discrimination of *Leishmania* species [138,139]. Some studies evaluated the 7SL RNA gene for species discrimination and although most species can be discriminated, it is not possible to discriminate *L*. (*L*.) *donovani*/*L*. (*L*.) *infantum*, *L*. (*V*.) *naiffi*/*L*. (*V*.) *braziliensis*, and *L*. (*L*.) *mexicana*/*L*. (*L*.) *pifanoi* by sequencing [134,139]. Several other genes encoding metabolism enzymes may also be used simultaneously for species discrimination (see below in Section 3.4.3. Multilocus Sequence Typing). These genes have not yet been evaluated individually and the potential of these genes for species discrimination is still unknown. These genes are single copy, limiting their direct detection in clinical samples.

For the kDNA, the only target already proven to be useful for species discrimination is the cytochrome B gene. Nucleotide sequence analysis revealed limitation for discrimination of *L*. (*L*.) *donovani*/*L*. (*L*.) *infantum* and *L*. (*V*.) *braziliensis*/*L*. (*V*.) *peruviana* [52]. Minicircles, another component of kDNA, may be useful for parasite detection due to the high number of copies, however, their variability within one strain/isolate prevents their use for species discrimination by DNA sequencing [54,121,140].

#### 3.4.3. Multilocus Sequence Typing (MLST)

This method uses the PCR method followed by DNA sequencing of some housekeeping genes (between 4 and 6) that are evaluated simultaneously. MLST increases the capacity of discrimination among different clinical samples, mainly in endemic regions. The main genes used in MLST analyses are aspartate aminotransferase (ASAT), isocitrate dehydrogenase (ICD), malic enzyme, mannose phosphate isomerase (MPI), 6-phosphogluconate dehydrogenase (6PGD), glucose-6-phosphate dehydrogenase (G6PD), glucose-6-phosphate isomerase (GPI), fumarate hydratase (FH), nucleoside hydrolase 1 (NH1), and nucleoside hydrolase 2 (NH2) [60,61,62,63]. Other genes that can be useful for *Leishmania* species typing by MLST are *hsp70*, miniexon, rDNA ITS1, and the 7SL RNA gene [134]. In general, this method has lower sensitivity than a single PCR method and requires optimization before implementation [102].

#### 3.4.4. Whole-Genome Sequencing (WGS)

Beyond studies on genomics and transcriptomics, for example, WGS by next generation technologies may also emerge as an alternative for identification and discrimination of different species of pathogens [141,142,143]. WGS was first applied for diagnosis in a sample of bone marrow aspirate from an immunosuppressed 61-year-old patient that confirmed an infection by *L*. (*L*.) *infantum*, probably acquired during a trip to Southern Italy [144]. This technology may also be useful for molecular surveillance of the disease, providing answers to key epidemiological questions of importance to public health, such as, for example: characterization of transmission cycles, detection of variants of the parasite with possible new clinical features, identification of genetic markers of clinical and epidemiological relevance, such as genes related to drug resistance and virulence, among others [145]. Although the cost is still the main limitation for this technology, WGS was also applied for the analysis of genomes of parasites from clinical samples of patients with VL due to *L*. (*L*.) *donovani* [146]. The main advantage of this technology is that it does not require parasite cultivation, thereby avoiding possible biological biases in the analyses. Variations in the genome and in the number of chromosome copies were found when these samples were compared to the genomic DNA of promastigotes cultivated in vitro [146].

#### 3.4.5. Real-Time PCR

Real-time PCR measures the amount of DNA generated by monitoring the amplification of a specific target during each PCR cycle. It has also been described as an alternative for diagnosis of leishmaniasis. Several methods have been implemented for detection, quantification of parasite burden and species typing, using different targets and protocols (reviewed by [107]). It has advantages including higher sensitivity, with a simpler standardization procedure, compared to standard PCR protocols. Moreover, there is no need for PCR product manipulation, since it is unnecessary to perform gel electrophoresis. As other PCR-based methods, real-time PCR protocols have been established using similar targets, such as kDNA, rDNA *locus*, *hsp70*, and other protein coding genes of the parasite (Table 1). Most of these protocols were performed using SYBR Green or TaqMan, while others were performed with MeltDoctor and LightCycler probes [107].

In patients with PKDL, real-time PCR was evaluated as a method for diagnosis and showed more than 90% sensitivity, while the sensitivity of microscopy was only 50.6% [147]. In an endemic area in Brazil, real-time PCR using SYBR Green or TaqMan was evaluated for diagnosis of CL and ML using two pair of primers targeting the kDNA of *L.* (*Viannia*) species, with 100% specificity and variation in sensitivity depending on the sample (biopsy or swab) and method used (ranging from 73.61% to 87.88%) [148]. In CL, specificity and sensitivity of detection may be affected by the method used for sample collection and must be selected appropriately [149]. For evaluation of parasite load by real-time PCR, the sort of lesion sample harvesting may also affect the quantification, with a higher detection in cytology brushes and scrapings samples when compared to biopsies, for example [25,150]. Real-time PCR applied to the investigation of parasite load in patients exposed to the treatment has already been evaluated in some studies. This can be useful for monitoring the efficacy of the treatment and identifying potential relapses, for example. In patients co-infected with HIV and VL, a significant decrease in the parasite burden was found in patients treated with amphotericin B, three months after the end of the treatment [151]. The parasite load could be used to predict cure and relapses with a sensitivity of 100% and specificity higher than 90% [151]. Similarly, monitoring of VL treatment through real-time PCR was also demonstrated in other studies using blood samples from patients. A consistent association between positive samples and relapsed patients was found, while patients that evolved to a clinical cure showed negative results in relation to the presence of the parasite DNA [152,153]. On the other hand, the application of real-time PCR for parasite loading in CL infections is more challenging throughout the treatment of the patients. First, in general, patients with ML had a lower parasite burden than localized CL infections, which can interfere with the sensitivity of the assay, as described in a SYBR green-based qPCR assay targeting kDNA to simultaneously detect and quantify *L.* (*Viannia*) species [154]. Depending on the causative species, the parasite loading may also be significantly different, with the highest burden in patients with severe ML disease [155]. Moreover, in patients that did not respond to treatment, a correlation with undetectable parasite loading was found, suggesting that the persistence of the parasite may be due to a limited access to the drugs or localization of the parasites in other tissues/locations not related with the primary infection; in this case the mucosal lesion [155]. Multiple infection genotypes associated with the same disease are also a challenge, since quiescent parasites and/or less sensitivity to the drugs, for example, may persist and consequently affect the determination of the parasite burden [156]. Real-time PCR was used for detection of CL in asymptomatic and subclinical infections using 7SL RNA as the target and for the determination of parasite viability [157]. The results demonstrated the technical feasibility to detect *L.* (*Viannia*) parasites in mucosal and blood monocyte samples from patients with subclinical infections [157]. Recently, patients’ urine was applied for molecular diagnosis by real-time PCR using samples of patients from Iran [158]. In this study, *Leishmania* DNA was detected and quantified in urine samples from patients with CL and VL, with a sensitivity of 89.2%. Previously, a similar sensitivity was described (88%) using urine samples of patients with VL and co-infected with HIV by conventional PCR [159].

For *Leishmania* species typing, real-time PCR was first applied by Weirather et al., [54]. In this study, authors evaluated a different combination of primers targeting kDNA minicircles and maxicircles and nuclear genes (in a total of 41 specific primer sets). A workflow with at least three serial real-time PCR was proposed, this being useful for identifying the main species responsible for VL and CL from serum and biopsy specimens respectively [54]. Although useful for discrimination of the main species endemic in Europe, Asia, and Africa, some species endemic in these regions were not evaluated, such as *L.* (*L.*) *aethiopica* in Africa and species of the subgenus *Viannia* autochthonous in the Amazon region: *L.* (*V.*) *naiffi*, *L.* (*V.*) *lainsoni*, *L.* (*V.*) *shawi*, and *L.* (*V.*) *lindenbergi*.

Some years later, a SYBR-Green assay targeting the ITS1 region of the parasite was validated for species discrimination, using reference strains and clinical isolates [53]. The proposed method was able to discriminate *Leishmania* in small groups containing 2–4 species, the main advantage being that this method can be accomplished by a single pair of primers, minimizing the risk of contamination [53]. This assay was incorporated into the CDC’s algorithm for diagnosis of leishmaniasis, and for identification of the definitive species, the ITS2-PCR followed by sequencing was proposed [53].

#### 3.4.6. PCR-High Resolution Melting (HRM)

PCR-HRM is a method based on variations in DNA sequences that uses double-stranded DNA binding dyes for measuring the intensity of fluorescence during dissociation of double-stranded to single-stranded DNA amplicons generated from a real-time PCR assay. This assay is performed with a new generation of saturating dyes (e.g., Eva Green or SYTO9) that needs specific equipment for PCR-HRM or an adapted real-time PCR instrument [107]. The method was established for discriminating the main species responsible for CL and VL in the Americas, Europe, Asia, and Africa. The main targets used for PCR-HRM were the *hsp70*, ITS1, and 7SL RNA genes [55,56,57,58]. Recently, this technology was also applied for identification and diagnosis of leishmaniasis using as a target an amino acid permease 3 (*aap3*) gene, an exclusive gene in trypanosomatids that is conserved in *Leishmania* [59]. The protocol was established using the main species responsible for CL and VL in the Americas, Europe, and Asia. The next goal for this technology is to implement it in endemic regions for its appropriated validation. It is a highly promising method, but requires an appropriate laboratory structure, with expensive equipment and trained professionals. Like other real-time PCR protocols, the results may be obtained quickly, with a reduced likelihood of contamination.

#### 3.4.7. Loop-Mediated Isothermal Amplification (LAMP)

This method is based on the amplification of DNA in less than one hour without the need for a thermocycler, this being a method already applied for other infectious diseases [160]. The amplified products can be detected visually using multiple parameters, including turbidity, fluorescence, and color with the naked eye and/or UV light [65]. LAMP is highly sensitive, with no postamplification handling or processing and may be implemented in endemic regions, with facilities requiring minimal structure for DNA extraction. It may be more sensitive than conventional PCR and has already been described as useful in the detection of *Leishmania* species and diagnosis of CL and VL (Table 1) [64,65,66]. Furthermore, this method may be used for the detection of RNA combining a reverse transcription step [9]. The main targets used are the 18S rRNA gene due to its high conservation in the genus *Leishmania* and elevated copy number, and minicircles, the main advantage of which is the high copy number per parasite, which increases the sensitivity of detection [65]. Recently, a LAMP assay targeting kDNA was described as able to detect the *L.* (*L.*) *donovani*, *L.* (*L.*) *major*, and *L.* (*L.*) *tropica* species [67]. The main limitation of LAMP is still the absence of a test able to discriminate different species of *Leishmania* in endemic regions where more than one species is present. A specific assay is only available for VL and/or PKDL due to *L.* (*L.*) *donovani* using kDNA minicircles as target [161].

## 4. Immunological Methods for Diagnosis of Leishmaniasis

Immunodiagnosis of leishmaniasis is based on the detection of antigens or anti-*Leishmania* antibodies in serum or urine samples from patients. Several immunological tests are available for diagnosis of leishmaniasis, particularly for VL, since it has a prominent humoral response [162]. In general, the methods used are: (i) Indirect fluorescent antibody test (IFAT); (ii) enzyme linked immunosorbent assay (ELISA); (iii) Western blot (or immunoblotting); (iv) direct agglutination test (DAT); (v) immunochromatographic strip test (ICT); and (vi) latex agglutination test (KAtex) (Table 1) [35,163]. The main challenges for immunological tests are cross-reactivity to other infectious diseases and false-positive results in some endemic areas [5,35]. Moreover, individuals co-infected with HIV can present negative results due to low antibody counts of the immunosuppressed condition [17,163,164]. Although serologic tests are available for CL, currently, they are not widely employed for CL diagnosis, considering that there is usually a poor humoral response from the host against parasites, meaning these tests exhibit low sensitivity [35]. New immunological tests are being developed to overcome this gap, using chemiluminescent ELISA to measure anti-α-galactosyl antibodies or the CL Detect Rapid Test targeting the peroxidoxin antigen of the parasite [2].

### 4.1. Leishmania Skin Test (LST)

Used for almost a century, the LST (also known as the Montenegro skin test) is based on a delay-type hypersensitivity response for total antigens of *Leishmania* promastigotes. It presents high sensitivity and specificity values (86–100% and >90%, respectively), being useful for epidemiological studies [165]. Results are determined 48–72 h post-injection and positivity is confirmed if induration has a diameter higher than 5 mm at the inoculation site of antigens (Table 1) [16]. Asymptomatic and symptomatic patients infected with CL generally present a positive result (positivity higher than 80%), while patients with DCL due to *L.* (*L.*) *amazonensis* have a LST negative result [16]. The test is also negative in patients with VL, becoming positive after successful treatment [166]. The major challenge for this test is its limitation in distinguishing present and previous infections, its use being restricted to people living in endemic areas.

### 4.2. Methods for Detection of Anti-Leishmania Antibodies

Methods for serological diagnosis have their sensitivity and specificity vary according to the method and antigens used, as well as to the host factors and the variations related to the species of *Leishmania* and the endemic region [23,50,89,97,167]. Serological tests have variable sensitivity due to antigenic differences in parasite species and low specificity due to cross-reactivity with other infectious diseases [168,169,170,171]. Serologic testing is recommended for VL patients when definitive diagnostic tests cannot be conducted or they have negative results [23]. The most accessible tests in routine practice for high income countries are ELISA, IFAT, indirect hemagglutination assay (IHA), and Western blot [27]. For this matter, recombinant proteins, such as the kinesin-related antigens rK39 and rK28, have been studied in order to develop faster and low-cost tests [172]. Moreover, serologic tests have a low sensitivity to VL diagnosis in HIV-infected patients and, therefore, tests for antileishmanial antibodies should not be performed as the only diagnostic assay, due to the potential for false-negative results [23,164]. Interestingly, recent studies have reported the potential use of urine for the detection of antibodies in VL, PKDL, and CL patients [158,173].

#### 4.2.1. Direct Agglutination Test (DAT)

The DAT is based on the agglutination reaction between antigen and antibody. For VL due to *L.* (*L.*) *infantum* and *L.* (*L.*) *donovani*, this test can be easily performed using serum or urine samples of patients [163]. This method showed high sensitivity (74.6–96.6%) and specificity (77.8–100%) in patients from Brazil, Spain, Kenya, Sudan, Ethiopia, and Nepal [50,71,86,87,90,91]. This test is also useful as marker of asymptomatic infection and in HIV co-infected individuals, showing sensitivity and specificity values of 81–91.3% and 83.3–90%, respectively [86,164,174,175]. Some limitations of this technique include the use of whole parasite antigen, which increases cross-reactivity with other infectious diseases, the need for serial dilutions, and a long incubation period [176]. Later, this method was improved for the Fast Agglutination Screening Test (FAST), with a shorter incubation period [177]. Another limitation is that this test remains positive up to one year after a clinical cure, so it cannot be used as a follow-up treatment, cure, or relapse [178]. Regarding diagnostic methods, DAT for CL using the *L.* (*L.*) *aethiopica* antigen showed high sensitivity (90.5%) and specificity (91.8%) in patients infected with this species; however, when the *L.* (*L.*) *donovani* antigen was used, a low sensitivity was found [179]. In Brazil, DAT and FAST were assayed to evaluate antibodies in patients with CL from an endemic area, but neither of these tests were useful for the diagnosis of this clinical form of the disease [180].

#### 4.2.2. Indirect Hemagglutination Assay (IHA)

The IHA uses sheep erythrocytes sensitized with a soluble *Leishmania* antigen, which are used for hemagglutination and for determining antibody titers [181]. It is considered sensitive and reliable in the detection of antileishmanial antibodies; however, it does not confirm the presence of parasites or parasite antigens, thus, it only indicates the exposure of the patient to the parasite [182]. It is well documented that individuals with VL may remain seropositive by IHA for periods of months or years; therefore, IHA is not sufficient as a sole diagnostic test for patients with clinically suspected VL [88]. Furthermore, this method has shown inferior performance in comparison with the microscopic detection of amastigotes in the bone marrow smear examination [182]. Sensitivity of IHA can vary according to the immune response of the host, for instance, the nutritional status can affect the immunoglobulin production rate. Poor nutritional status can lead to a low immune response and, hence, a low rate of immunoglobulins, which could explain the low IHA titer in these patients and false-negative results. Additionally, cross-reactions may be considered in regions where there are other endemic diseases, such as leprosy, Chagas disease, malaria, and schistosomiasis [183].

#### 4.2.3. Indirect Fluorescent Antibody Test (IFAT)

The main advantage of the IFAT is the possibility to discriminate between a cure and a possible relapse in VL. This test does not detect antibodies in VL patients’ samples after a cure and the persistence of antibodies in the serum indicates a possible relapse [24]. Its sensitivity and specificity are variable and the values are 28.4–92% and 83.3–94.4%, respectively, in immunocompetent patients from different endemic areas (Table 1) [71,86,87,88]. Meanwhile, in a meta-analysis study with HIV/VL co-infected patients, this test demonstrated highly variable values, with sensitivity and specificity of 11–82% and 81–99%, respectively [164]. Later, an in-house IFAT using antigens prepared from promastigotes of *L.* (*L.*) *infantum* showed a sensitivity of 79.4% and a specificity of 99.2% with serum of HIV/VL patients from Spain [86].

In patients from Brazil with ML, IFAT showed a sensitivity of 56.7% [184]. In another study conducted with Brazilian patients with CL or ML, it achieved sensitivity and specificity levels of 95.4% and 77.7%, respectively, when *L.* (*L.*) *major* promastigote antigens were used, with *L.* (*V.*) *braziliensis* promastigotes being used as antigens, it presented a sensitivity of 81.5% and a specificity of 86.2% [89].

#### 4.2.4. Enzyme Linked Immunosorbent Assay (ELISA)

The ELISA sensitivity and specificity vary depending upon the antigen used [176]. The advantage of ELISA is that it can be used for a large number of samples with different antigens and different types of samples at the same time [163]. On the other hand, the disadvantages include the time necessary, the need for specialized professionals, sophisticated equipment, and the lack of discrimination between an active disease and the clinical cure, with its use being limited in endemic regions [176].

For VL diagnosis, the rK39 antigen is mostly used, a 39-amino acid containing a kinesin-related peptide of *L.* (*L.*) *infantum* [185]. The sensitivity and specificity of ELISA-rK39 vary around 88.6–99% and 81–98.2%, respectively, in endemic regions for VL due to *L.* (*L.*) *infantum* and *L.* (*L.*) *donovani* [68,69,70,71,72,73]. In patients infected by *L.* (*L.*) *infantum* who are co-infected with HIV, the rK39 antigen had a sensitivity of 82% [186]. Other recombinant proteins, such as rK26, rKRP42, rK9, rKE16, rA2, and rKDDR, among others, were also tested and showed varied sensitivity in VL patients [74,75,76,77,78]. The BHUP1 antigen, a heat shock protein, showed 95% sensitivity and 96–100% specificity, using sera from untreated and successfully treated patients from a VL endemic area in India [82]. Other antigens of this same family of proteins, BHUP2 and BHUP3, also showed satisfactory sensitivity (88–94%) and specificity (96–100%) for diagnosis of VL [81,83]. The heat shock proteins HSP70 and HSP83 proved to be good candidates for VL diagnosis. In the case of rHSP83, it is recognized in sera of CL, ML, and VL patients, with an insignificant cross-reaction with other diseases [79,80]. In a recent study, a hypothetical protein from *L.* (*L.*) *infantum* (LiHyE) showed good results in terms of sensitivity in samples from patients with CL and VL, when compared to other antigens. This recombinant protein was considered to be a promising marker for differentiating healthy individuals from those infected with CL or VL [187,188].

As an alternative for the detection of antibodies in the serum, a urine ELISA assay was developed for diagnosis of anti-*Leishmania* antibodies. For VL, the sensitivity and specificity of this assay were 97.94% and 100%, respectively, while for PKDL, sensitivity and specificity were 100% [158]. This assay is based on a *Leishmania* promastigote membrane antigen that showed the best recognition of IgG antibodies compared to other tested antigens [recombinant GP63 and CPA (cysteine protease) proteins and soluble leishmanial antigens from *L.* (*L.*) *donovani*] [158].

In patients with ML, ELISA presented a sensitivity of 93.3% using the total antigen of *L.* (*L.*) *major* promastigotes [184], whereas amongst patients with CL, the sensitivity of this same test was only 66.3% [167]. In patients with CL caused by *L.* (*L.*) *tropica*, ELISA using total antigens of *L.* (*L.*) *infantum* promastigotes, showed sensitivity and specificity of 78% and 95.3%, respectively [97]. ELISA serology in Brazilian CL/HIV co-infected patients showed a sensitivity of 77% [189]. Sensitivity of 95.7% and specificity of 100% was achieved using *L.* (*V.*) *braziliensis* total antigens, while with *L.* (*L.*) *major*, lower values of sensitivity (78.7%) and specificity were observed (78.7% and 82.8%, respectively) for diagnosis of Brazilian patients with CL and ML [89]. Alternative antigens, such as HSP60 of *L.* (*L.*) *major* and HSP70 of *L.* (*V.*) *braziliensis*, were shown to be good candidates, although cross-reactivity may occur with the serum of patients with VL [190,191]. Recombinant proteins of *L.* (*L.*) *infantum*, such as HSP70, histone proteins, and a protein of the kinetoplast (KMP11), were also tested using sera from patients with CL, with HSP70 showing the best performance [170]. Another study using the recombinant version of the HSP83.1 protein from *L.* (*V.*) *braziliensis* showed that it is a promising antigen for the immunodiagnosis of both clinical forms of leishmaniasis [192].

Recombinant proteins from *L.* (*V.*) *braziliensis* were evaluated for CL diagnosis, using serum from patients with CL and ML [84,85]. Among the several proteins identified as antigenic, tryparedoxin peroxidase showed the best result, with 100% sensitivity and specificity [84]. The reactivity of rLb8E and rLb6H antigens from *L.* (*V.*) *braziliensis* was evaluated in CL patients [85]. Both antigens showed good performance, however, rLb6H showed greater reactivity with *L.* (*L.*) *amazonensis*, followed by *L.* (*V.*) *braziliensis* and *L.* (*V.*) *guyanensis*. Other recombinant proteins, such as LiHypA, SMP-3, CcOx, HRF, rLiHyL, and others, were evaluated and presented good results, with some studies observing good sensitivity and specificity for both CL and VL [187,193,194,195]. In general, these recombinant proteins have higher sensitivity and specificity than the soluble *Leishmania* antigen extract, but they do not discriminate CL and VL infection.

#### 4.2.5. Immuno-Chromatographic Test (ICT) (Strip Test)

The ICT using rK39 antigen is widely used in the diagnosis of VL, however its sensitivity and specificity may vary depending on the product and patient’s origin, as demonstrated in studies with patients from different endemic areas in Brazil, Spain, East Africa, and Southeast Asia (Table 1) [50,70,86,90,92,196]. In HIV positive patients from Spain, 67.3% sensitivity and 100% specificity were observed [86].

The advantage of this test includes low cost, rapid, easy and simple performance. Similar to ELISA, this test does not discriminate between the active disease and the clinical cure, restricting its use in endemic regions [176]. Other recombinant antigens, such as rK28, were evaluated for the immunochromatographic assay (or lateral flow immunoassay) [197,198,199,200]. As an alternative, a dipstick test containing *L.* (*L.*) *donovani* membrane antigens was developed, presenting sensitivity and specificity of 100% in Indian and Brazilian patients with VL (Table 1) [94]. This test was evaluated later for VL, using sera from more than 1000 subjects from eight centers in six endemic countries (India, Nepal, Sri Lanka, Brazil, Ethiopia, and Spain) [93]. The overall sensitivity and specificity for all these regions were 97.1% and 93.44%, respectively, and a better performance of this test was found compared to the rK39 rapid test (see below) in these regions [93]. More important, the dipstick test did not cross react with serum samples of patients with CL. Previously a dipstick test was described using this same antigen for detection of antibodies in the urine of patients with VL and PKDL with a sensitivity of 100% [173]. The main membrane antigens recognized by serum from VL patients were characterized by mass spectrometry and included the receptor of activated C kinase (LACK), beta-tubulin isoforms, ATP synthase α-chain, elongation factor 1-α (EF1-α), GP63, HSP70, and nucleoporins-93 (NUP-93) [201,202,203,204].

More recently, laser direct-write technology was used for the development of a new lateral flow immunoassay device containing the recombinant proteins β-tubulin and LiHyp1. This device is less expensive than other immuno-chromatographic tests and presented high sensitivity (90.9%) and specificity (98.7%) using blood/serum from patients diagnosed with VL [205].

#### 4.2.6. rK39 Rapid Diagnostic Test (RDT)

The K39 protein provides the basis for the developed rK39 RDT. The result arrives between 10 and 20 min in the form of a binary reading (positive or negative), with sensitivity estimated at 97% in India and 85% in Eastern Africa [206]. The rK39 RDT is the most widely used tool and the first choice for a decentralized diagnosis of VL in endemic areas, but it cannot discriminate between current, subclinical, or past infections [207]. This test was applied for evaluation of *Leishmania* asymptomatic infection in HIV patients from an endemic area in Ethiopia [208]. The rK39 RDT was considered the most common marker for asymptomatic infection in these patients, compared to DAT and KAtex (see below), and real-time PCR [209]. In the northeastern region of Brazil, the sensitivity in VL/HIV co-infected patients was low when compared with patients with VL and sensitivity was 0.0% and 25% using oral fluid and serum/whole blood, respectively, while for VL patients the sensitivity ranged from 80% to 96.3% using whole blood and serum and 43.3% to 88.9% in oral fluid, depending on the origin of these Brazilian patients [210]. This variation in sensitivity may be explained by the extensive kinesin genetic diversity in strains and isolates of *Leishmania*, as described for different *L.* (*L.*) *donovani* strains from East Africa and South Asia [209]. These findings highlight the importance of considering the use of this test in particular endemic areas.

#### 4.2.7. Western Blot

Western blot has high sensitivity (98%) and specificity (100%) and can be more sensitive than IFAT and ELISA (Table 1) [95,96,97]. In HIV/*Leishmania* co-infected patients the sensitivity and specificity were 84% and 82%, respectively [164]. Serum of CL patients due to *L.* (*L.*) *tropica* infection detected proteins of 15–118 kDa of an antigenic extract of *L.* (*L.*) *infantum* promastigotes [97]. Among these proteins, a band of 63 kDa had the highest sensitivity (99.1%) [97]. Recently, serum samples from VL patients were used to detect 34 protein bands by Western blot using *L.* (*L.*) *infantum* promastigotes extract [98]. In addition to this, urine from CL and VL patients from Iran was recently described as useful for detection of antibodies against the parasite by Western blot using *L.* (*L.*) *infantum* extract, with sensitivity 78.2% and 92.8% for CL and VL, respectively [158]. The main advantage of Western blot is the detailed antibodies’ responses to various antigens. However, it is a time consuming and expensive process, besides needing qualified professionals [176].

### 4.3. Methods for Detection of Antigens of Leishmania

Antigen detection can be considered more specific than antibody-based immunodiagnostic tests and is also useful in the diagnosis of the disease in cases where there is deficient antibody production (as in HIV patients) [211]. A latex test (KAtex) based on detection of a heat-stable low molecular-weight carbohydrate antigen in urine samples has been developed [165]. This KAtex assay showed varying sensitivity (35.8%–100%) and specificity (64%–98.3%), depending on the endemic region (Table 1) [49,50,51]. Interestingly, more than 90% of cured patients had a negative KAtex result [51], indicating that this method may discriminate current disease and clinical cure of VL. In addition, in HIV/*Leishmania* co-infected individuals, this test showed good sensitivity (85.7%–100%) [211,212,213]. The test is simple, easy to perform, inexpensive, quick, and can be used as a screening test, since specificity can be high, but it has low to moderate sensitivity [176]. Furthermore, it also contributes to improving VL diagnosis and monitoring treatment response in HIV co-infected patients, reducing the exposure to invasive procedures amongst these vulnerable individuals [214].

Other antigens were previously identified in the urine (Li-isd1, Li-txn-1, and Li-ntf2) of VL patients infected with *L.* (*L.*) *infantum*. These antigens were used as recombinant proteins for the production of antibodies and to develop a capture ELISA for detection of these proteins in the urine of VL patients. This method presented sensitivity and specificity of 89% and 100%, respectively, and a limit of detection of 4–10 pg of antigen per mL of urine [215,216]. This assay was improved by including other three proteins (Ld-mao1, Ld-ppi1, and Ld-mad1) for the production of an antigen detection capture ELISA for diagnosis of VL caused by *L.* (*L.*) *infantum* and *L.* (*L.*) *donovani* [217]. This multiplexed capture ELISA based on monoclonal antibodies is able to simultaneously detect these six proteins in the urine of patients and had a sensitivity higher than 93% [217]. This test did not cross-react with the urine of healthy individuals or of patients with non-VL tropical diseases, and it has been proposed that it should be validated in clinical use [217]. Recently another six proteins were identified by mass spectrometry (40S ribosomal protein S9, two protein kinases, and another four hypothetical proteins) from urine samples of Indian and Sudanese VL patients, being used to predict epitope regions highly specific to *Leishmania* spp. and suitable for raising antibodies for the development of an antigen capture assay [218].

## 5. Conclusions

Several methods and tools have been developed over recent years for the detection, quantification, and identification of the parasite of the genus *Leishmania*. Although advances in these methods have improved the sensitivity and specificity of leishmaniasis diagnosis, there are still some challenges to be overcome. For instance, developing affordable, fast, and accessible tests that can define *Leishmania* species will be a turning point in diagnosis, since the discrimination of species has significant importance for prognosis and species-specific treatments. Parasitological and microscopic examinations are broadly used; although highly specific, they present insufficient sensitivity and do not provide *Leishmania* species identification. Furthermore, biopsies can be very invasive and life-threatening, such as in some patients with VL. In vitro and in vivo cultivation are rarely used in routine clinical practice, given that they are generally only available in leishmaniasis diagnosis reference centers and the results can take weeks to be delivered. To fill the gap of non-invasive tests and define *Leishmania* species, molecular tests are promising and already available, but remain reserved for reference laboratories and are usually found only in high-income countries. Alternatively, rapid tests are promising to deliver non-invasive and low-priced methods, but there are still limitations in endemic regions due to false positives. Therefore, there is a need for a simple, fast, and accurate test with high sensitivity and specificity, which can be used without any specific expertise, considering the conditions that prevail in endemic areas, where sophisticated methods cannot be employed.

## Figures and Tables

**Table 1:** Summary of the main methods used for diagnosis of CL and VL.

	Diagnostic Method	Clinical Form	Culture Required	Species Discrimination ^1^	Reference(s)
Parasitological methods	Biopsy, punch, scraping, smear or imprinting followed by microscopic examination	CL	No	No (only genus *Leishmania*)	[25,26,29,30]
	Bone marrow, lymph nodes or spleen aspirates or liver biopsy followed by microscopic examination	VL	No	No (only genus *Leishmania*)	[33,34]
	In vitro cultivation	VL, CL	Yes	No (only genus *Leishmania*)	[37]
	Inoculation in animals (mice or hamsters)	VL, CL	No	No (only genus *Leishmania*)	[22,35]
	Xenodiagnosis	VL	No	No (only genus *Leishmania*)	[39,40]
Protein-based methods	MLEE	VL, CL	Yes	Yes (almost all currently identified species)	[41]
	Monoclonal antibodies	VL, CL	Yes	Yes (almost all species endemic in the Americas and also *L.* (*L.*) *major, L.* (*L.*) *donovani* and *L.* (*L.*) *tropica*)	[42,43,44,45]
	MALDI-TOF MS	VL, CL	Yes	Yes (all species endemic in the Americas, Europe, Asia and Africa)	[46,47,48]
	KAtex	VL	No	No (only genus *Leishmania*)	[49,50,51]
DNA-based methods	PCR-RFLP	VL, CL	No	Yes (almost all species; depends on the target)	[9,52]
	DNA sequencing	VL, CL	No	Yes (almost all species; depends on the target)	[9,52]
	Real-time PCR	VL, CL	No	Yes (most species)	[53,54]
	PCR-HRM	VL, CL	No	Yes (almost all species endemic in the Americas, Europe, Asia and Africa)	[55,56,57,58,59]
	MLST	VL, CL	No	Yes (all species)	[60,61,62,63]
	LAMP	VL, CL	No	Yes (limited to certain species)	[64,65,66,67]
Immunological-based methods	*Leishmania* skin test	CL (negative for DCL)	No	No (only genus *Leishmania*)	[16]
	ELISA (rK39)	VL	No	No (only genus *Leishmania*)	[68,69,70,71,72,73]
	ELISA (other recombinant antigens)	VL, CL	No	No (only genus *Leishmania*)	[74,75,76,77,78,79,80,81,82,83,84,85]
	IFAT	VL, CL	No	No (only genus *Leishmania*)	[71,86,87,88,89]
	DAT	VL	No	No (only genus *Leishmania*)	[50,71,86,87,90,91]
	ICT (rK39)	VL	No	No (only genus *Leishmania*)	[50,70,86,90,92]
	Dipstick test [*L.* (*L.*) *donovani* promastigote antigens]	VL	No	No (only genus *Leishmania*)	[93,94]
	Western blot	VL, CL	No	No (only genus *Leishmania*)	[95,96,97,98]

^1^ Check in the text and references for details of species evaluated in the respective methods.
